# Projections of psychiatrists’ distribution for patients in Japan: a utilization-based approach

**DOI:** 10.1186/s12960-021-00594-z

**Published:** 2021-04-09

**Authors:** Norio Sugawara, Norio Yasui-Furukori, Kazutaka Shimoda

**Affiliations:** Department of Psychiatry, Dokkyo Medical University School of Medicine, 880 Kitakobayashi, Mibu, Tochigi 321-0293 Japan

**Keywords:** Forecasting, Demand–supply balance, Maldistribution, Utilization-based approach

## Abstract

**Background:**

Depopulation accompanied by population aging is a major public health concern in Japan. Although adequate allocation of mental healthcare resources is needed, there have been few studies on the impact of population change on the supply–demand balance for mental illness in Japan. The aim of this study is to predict psychiatrists' distribution for patients with mental illness via a utilization-based approach.

**Methods:**

We set patients with schizophrenia, mood disorders, vascular dementia or Alzheimer’s disease as study subjects and conducted analyses for 2015, 2025, 2035, and 2045 across all prefectures. Moreover, we evaluated the regional maldistribution of demand and supply by calculating the number of psychiatrists per patient, Gini coefficients (GC), and Herfindahl–Hirschman Index (HHI).

**Results:**

The mean number of psychiatrists per patient for patients with schizophrenia, mood disorders, vascular dementia, and Alzheimer’s disease in 2025, 2035, and 2045 was significantly lower than in 2015. For all of the abovementioned diseases, both the GC and HHI will increase until 2045.

**Conclusion:**

If psychiatrists are allocated at the current population-to-psychiatrist ratio, the shortage of psychiatrists will continue to worsen in the future. To overcome this inequity, policy makers should make plans to shift responsibilities from psychiatrists to other mental health workers and to ensure the adequate geographical allocation of healthcare resources.

## Introduction

The increasing number of patients with mood disorders and Alzheimer's disease has increased the demand for psychiatrists in Japan [1]. In 2013, the Ministry of Health, Labour, and Welfare (MHLW) designated mental illness as the fifth priority disease for the national medical service, and all prefectures in Japan were required to start regional medical care planning for mental illness [2]. Optimizing the balance between supply and demand for the mental healthcare system is a public health issue, and psychiatrists are an essential human resource for the system.

Healthcare systems in Japan are facing the problems of depopulation accompanied by population aging. Based on 2015 national census data, the National Institute of Population and Social Security Research (IPSS) predicted that the Japanese population will decrease from 127 to 106 million by 2045 [3]. In the same analysis, the IPSS also indicated that the elderly population (aged 65 years and over) will increase by 15.7%, while the young population (aged 0 to 14 years) will decrease by 28.6%. Because the age of onset differ**s** by disease, changes in population structure could lead to different utilization patterns of healthcare services for each mental illness. Although adequate allocation of mental healthcare resources is needed, there have been few studies concerning the impact of population change on the supply–demand balance with respect to mental illness in Japan.

In this study, we employed a utilization-based approach in which current or target rates of healthcare system utilization are multiplied by future population estimates to estimate the demand of mental illness. This approach has been widely used in Organisation for Economic Co-operation and Development (OECD) member countries [4, 5]. There have been other approaches to estimate the demand for healthcare workforces. A service-based approach or a task-based approach could be used for the same purpose [6]. The former approach is based on the estimation of the shifting needs of an organization that are required to operate effectively. The estimation requires data on the burden of disease, epidemiological changes, hospital bed-to-staff ratios, and the expected budget for staff salaries. On the other hand, the estimation of need in a task-based approach is founded on the tasks a typical professional can undertake in a given time period. Both approaches could be useful for healthcare workforce planning at relatively local level, but effectively accomplish this planning at the national level requires an enormous amount of data.

The aim of this study is to predict psychiatrists' distribution for patients with mental illness and to predict the future healthcare supply–demand balance. The projections of the availability of human resources in the mental healthcare system could support policy decision-making. To the best of our knowledge, this study is the first report on the projection of psychiatrists’ distribution for patients with mental illness in Japan.

## Methods

### Analytical parameters

Data on the number of psychiatrists per prefecture were obtained from the 2016 Survey of Physicians, Dentists, and Pharmacists (SPDP) on the MHLW website [7]. In addition, we obtained data on population projections until 2045 from the IPSS [3]. These projections took the count from the 2015 Population Census as the base population [8]. The utilization rate per 100,000 population per prefecture was obtained from the 2017 Patient Survey on the MHLW website [1]. Based on the Statistics Act, Article 2, the MHLW conducted the Patient Survey to obtain basic data needed for the development of health policies by identifying age, sex, diagnosis according to the International Classification of Diseases, tenth revision (ICD-10), and condition at time of survey for each patient. The survey covers inpatient and outpatient treatment in medical facilities, including 6395 hospitals and 5526 clinics. The survey report provides estimates of the utilization rates broken down by sex, 5-year age groups and ICD-10 diagnoses. We set patients with schizophrenia, mood disorders, vascular dementia, and Alzheimer’s disease as the study subjects, as those are the leading mental disorders associated with relatively higher loss of disability-adjusted life-years (DALYs) in Japan [9]. The requirement for written informed consent was waived by the Ethics Committee since the study involved record review only.

To project the number of future psychiatrists, we assumed that the psychiatrist-to-population ratio in Japan would be constant from 2015 to 2045. Although the current distribution of psychiatrists in Japan is not adequate, we assumed that attractiveness of urban areas to psychiatrists would not change [10]. We calculated the number of psychiatrists per population based on the 2016 SPDP and the 2015 Population Census for each prefecture. Following the abovementioned assumption, the estimation of the number of psychiatrists in 2015, 2025, 2035 and 2045 was based on the psychiatrist-to-population ratio and population projections in Japan.

In a utilization-based approach, future demand is calculated by multiplying the future population by the utilization rate for each disease. First, we obtained the utilization rate for each disease, by age and sex, as variables from the 2017 Patient Survey. Population by age and sex in the future was based on population projections from 2015 to 2045. We then multiplied these variables for estimates of the future number of patients as the criterion for demand in each prefecture. For each disease, we calculated the number of psychiatrists per patient in Japan.

We employed the Gini coefficient (GC) as an indicator of the distribution of psychiatrists to aid in the evaluation of inequity in human resources by prefecture. In this study, Lorenz curves are drawn by plotting the cumulative proportion of psychiatrists on the vertical axis and the cumulative proportion of the estimated number of patients on the horizontal axis in ascending order by psychiatrists per patient across all prefectures. After that, we calculated the GCs based on the Lorenz curves. The GC is traditionally used to analyze the distribution of income and wealth and has a theoretical range from 0 (perfect evenness) to 1 (maximum possible unevenness). It provides a standardized value to reflect the relative unevenness of a distribution. In this study, higher values of the GC indicated higher levels of human resource (psychiatrists) inequality experienced by patients with mental illnesses among prefectures.

The Herfindahl–Hirschman Index (HHI), which has been widely used to evaluate mergers and acquisitions, was adopted as an indicator of patient concentration. In this study, the HHI for each disease is calculated as the sum of squared patient shares (percentages) across all prefectures. It approaches zero when a market is occupied by a large number of competitors of relatively equal size and reaches its maximum of 10,000 points when there is a market monopoly. The HHI was interpreted as the concentration of patients with mental illness to estimate future demand transfer. In this study, higher values of the HHI indicated higher concentrations of patients with mental illnesses among prefectures.

In Tables 1 and 2, we ordered the prefectures according to the identification codes (JIS X 0401) from the Japanese Industrial Standards Committee.

**Table 1:** Forecasted psychiatrists and patients, 2015 to 2045

	Psychiatrists				Schizophrenia				Mood disorders				Alzheimer's disease				Vascular dementia and others			
	2015	2025	2035	2045	2015	2025	2035	2045	2015	2025	2035	2045	2015	2025	2035	2045	2015	2025	2035	2045
Hokkaido	733	683	619	545	9569	9071	8297	7303	5194	4998	4537	3993	4227	5647	6804	6744	1725	2313	2753	2819
Aomori	153	135	116	96	2371	2160	1877	1562	1275	1164	1013	838	1089	1357	1547	1530	441	556	628	632
Iwate	125	113	100	86	2283	2118	1896	1641	1233	1144	1021	881	1146	1349	1485	1461	463	555	607	611
Miyagi	266	254	233	206	3955	3894	3656	3275	2177	2143	1993	1763	1645	2157	2624	2717	673	894	1067	1133
Akita	142	123	103	84	1912	1685	1419	1154	1020	904	769	618	1043	1213	1316	1262	418	498	533	521
Yamagata	146	132	117	100	1989	1827	1626	1401	1076	991	887	761	1079	1220	1341	1330	435	508	547	551
Fukushima	209	189	167	144	3388	3167	2831	2430	1821	1702	1525	1307	1614	1947	2260	2309	656	810	922	964
Ibaraki	241	227	208	185	5047	4871	4518	4035	2722	2667	2446	2184	1999	2664	3277	3297	831	1103	1333	1381
Tochigi	176	167	154	139	3398	3288	3077	2788	1839	1799	1675	1507	1343	1706	2083	2104	554	709	846	885
Gunma	232	219	202	183	3400	3280	3076	2782	1841	1804	1667	1511	1463	1883	2261	2247	603	779	922	946
Saitama	617	612	587	554	12,333	12,430	12,168	11,475	6747	6932	6612	6277	3984	6185	7754	7881	1682	2524	3166	3322
Chiba	624	614	584	548	10,672	10,612	10,279	9629	5821	5911	5594	5269	3703	5551	6851	6840	1553	2266	2798	2885
Tokyo	2057	2107	2108	2071	22,127	23,146	23,785	23,404	12,688	13,237	13,173	13,040	7822	10,612	12,467	13,286	3218	4348	5158	5597
Kanagawa	989	983	948	901	15,189	15,468	15,260	14,374	8470	8677	8321	7957	5236	7848	9665	10,052	2183	3212	3981	4238
Niigata	217	201	181	160	4051	3793	3455	3057	2196	2077	1883	1660	2045	2443	2773	2721	830	1013	1129	1141
Toyama	133	124	114	102	1880	1772	1639	1474	1015	978	891	800	913	1133	1307	1227	373	467	530	517
Ishikawa	163	156	146	134	1965	1898	1793	1646	1072	1057	984	905	875	1109	1329	1296	359	456	540	544
Fukui	92	86	79	72	1351	1279	1190	1071	734	705	654	589	660	786	907	896	268	326	369	375
Yamanashi	92	84	75	66	1443	1364	1235	1069	788	743	668	586	688	842	981	990	281	349	400	412
Nagano	228	213	195	175	3630	3458	3218	2883	1977	1899	1739	1570	1896	2283	2599	2568	775	946	1066	1079
Gifu	173	162	148	133	3474	3302	3062	2740	1894	1825	1663	1494	1514	1947	2260	2202	624	798	921	924
Shizuoka	342	324	300	272	6400	6142	5737	5183	3475	3385	3122	2837	2722	3635	4320	4304	1121	1495	1766	1810
Aichi	760	757	734	701	12,215	12,407	12,321	11,787	6807	6992	6754	6490	4257	6141	7486	7685	1772	2511	3068	3240
Mie	219	206	190	173	3111	2985	2790	2524	1701	1649	1514	1379	1367	1729	1994	1978	562	709	813	826
Shiga	128	126	122	114	2295	2314	2266	2139	1271	1296	1245	1178	892	1191	1474	1525	370	493	603	642
Kyoto	353	339	316	289	4399	4267	4053	3712	2441	2415	2240	2063	1852	2544	3039	2952	761	1036	1235	1238
Osaka	1052	1015	948	873	14,928	14,577	13,952	12,859	8275	8245	7650	7122	5454	7970	9382	9156	2262	3228	3834	3855
Hyogo	590	566	528	483	9460	9242	8788	8033	5192	5144	4794	4421	3832	5283	6377	6394	1577	2160	2597	2677
Nara	161	149	134	118	2365	2218	2010	1755	1286	1236	1103	973	993	1361	1633	1572	413	557	663	657
Wakayama	102	93	83	73	1705	1561	1401	1228	927	861	766	674	837	986	1085	1026	341	405	440	426
Tottori	96	90	83	75	995	933	859	778	540	516	475	426	529	613	697	691	213	255	284	289
Shimane	117	108	99	89	1227	1128	1030	927	661	620	567	506	718	807	881	836	290	335	358	350
Okayama	296	284	268	250	3244	3125	2976	2778	1784	1757	1654	1537	1556	1935	2234	2167	635	794	911	910
Hiroshima	370	359	339	316	4812	4667	4446	4137	2644	2619	2461	2287	2136	2768	3264	3177	878	1139	1331	1338
Yamaguchi	202	186	168	149	2494	2279	2059	1838	1345	1263	1132	1004	1282	1554	1740	1603	522	637	704	674
Tokushima	131	119	106	93	1345	1236	1108	969	727	677	604	525	683	790	889	851	278	328	361	356
Kagawa	142	134	124	113	1704	1610	1503	1369	921	890	823	745	834	1001	1155	1108	341	416	470	466
Ehime	141	130	117	103	2446	2273	2061	1824	1323	1246	1126	992	1230	1479	1691	1631	499	608	684	683
Kochi	123	110	97	84	1305	1173	1039	899	707	647	567	489	731	830	912	847	294	339	369	354
Fukuoka	856	846	812	764	8545	8511	8247	7777	4728	4777	4588	4300	3541	4713	5772	5858	1450	1936	2339	2451
Saga	161	152	141	128	1412	1342	1244	1132	768	742	693	624	692	818	945	958	280	339	382	396
Nagasaki	219	200	179	156	2420	2233	1990	1733	1308	1216	1092	952	1213	1440	1640	1620	492	594	664	675
Kumamoto	335	317	296	271	3047	2893	2677	2436	1667	1600	1491	1353	1569	1861	2126	2136	635	772	865	889
Oita	181	169	155	139	2036	1907	1738	1564	1107	1050	963	860	1041	1266	1456	1406	423	522	591	590
Miyazaki	191	177	161	143	1918	1786	1605	1425	1038	977	890	784	961	1170	1355	1346	391	483	551	558
Kagoshima	265	243	219	194	2859	2659	2386	2101	1554	1441	1312	1151	1522	1730	1949	1987	615	722	793	826
Okinawa	268	274	274	267	2199	2339	2379	2315	1239	1295	1310	1277	793	1065	1350	1561	328	448	557	653
Mean	332	320**	302**	279**	4602	4504**	4298**	3966**	2533	2509*	2354**	2180**	1898	2523**	2994**	3007**	781	1036**	1222**	1262**

The Wilcoxon Signed-Ranks Test with the Bonferroni correction was employed for comparisons between 2015 and other time points

*p < 0.05, **p < 0.001

**Table 2:** Forecasted psychiatrist per patients, 2015–2045

	Schizophrenia				Mood disorders				Alzheimer's disease				Vascular dementia and others			
	2015	2025	2035	2045	2015	2025	2035	2045	2015	2025	2035	2045	2015	2025	2035	2045
Hokkaido	0.077	0.075	0.075	0.075	0.141	0.137	0.136	0.136	0.173	0.121	0.091	0.081	0.425	0.295	0.225	0.193
Aomori	0.065	0.063	0.062	0.061	0.120	0.116	0.115	0.115	0.140	0.099	0.075	0.063	0.347	0.243	0.185	0.152
Iwate	0.055	0.053	0.053	0.052	0.101	0.099	0.098	0.098	0.109	0.084	0.067	0.059	0.270	0.204	0.165	0.141
Miyagi	0.067	0.065	0.064	0.063	0.122	0.119	0.117	0.117	0.162	0.118	0.089	0.076	0.395	0.284	0.218	0.182
Akita	0.074	0.073	0.073	0.073	0.139	0.136	0.134	0.136	0.136	0.101	0.078	0.067	0.340	0.247	0.193	0.161
Yamagata	0.073	0.072	0.072	0.071	0.136	0.133	0.132	0.131	0.135	0.108	0.087	0.075	0.336	0.260	0.214	0.181
Fukushima	0.062	0.060	0.059	0.059	0.115	0.111	0.110	0.110	0.129	0.097	0.074	0.062	0.319	0.233	0.181	0.149
Ibaraki	0.048	0.047	0.046	0.046	0.089	0.085	0.085	0.085	0.121	0.085	0.063	0.056	0.290	0.206	0.156	0.134
Tochigi	0.052	0.051	0.050	0.050	0.096	0.093	0.092	0.092	0.131	0.098	0.074	0.066	0.318	0.236	0.182	0.157
Gunma	0.068	0.067	0.066	0.066	0.126	0.121	0.121	0.121	0.159	0.116	0.089	0.081	0.385	0.281	0.219	0.193
Saitama	0.050	0.049	0.048	0.048	0.091	0.088	0.089	0.088	0.155	0.099	0.076	0.070	0.367	0.242	0.185	0.167
Chiba	0.058	0.058	0.057	0.057	0.107	0.104	0.104	0.104	0.169	0.111	0.085	0.080	0.402	0.271	0.209	0.190
Tokyo	0.093	0.091	0.089	0.088	0.162	0.159	0.160	0.159	0.263	0.199	0.169	0.156	0.639	0.485	0.409	0.370
Kanagawa	0.065	0.064	0.062	0.063	0.117	0.113	0.114	0.113	0.189	0.125	0.098	0.090	0.453	0.306	0.238	0.213
Niigata	0.054	0.053	0.052	0.052	0.099	0.097	0.096	0.096	0.106	0.082	0.065	0.059	0.261	0.198	0.160	0.140
Toyama	0.071	0.070	0.070	0.069	0.131	0.127	0.128	0.128	0.146	0.109	0.087	0.083	0.357	0.266	0.215	0.197
Ishikawa	0.083	0.082	0.081	0.081	0.152	0.148	0.148	0.148	0.186	0.141	0.110	0.103	0.454	0.342	0.270	0.246
Fukui	0.068	0.067	0.066	0.067	0.125	0.122	0.121	0.122	0.139	0.109	0.087	0.080	0.343	0.264	0.214	0.192
Yamanashi	0.064	0.062	0.061	0.062	0.117	0.113	0.112	0.113	0.134	0.100	0.076	0.067	0.327	0.241	0.188	0.160
Nagano	0.063	0.062	0.061	0.061	0.115	0.112	0.112	0.111	0.120	0.093	0.075	0.068	0.294	0.225	0.183	0.162
Gifu	0.050	0.049	0.048	0.049	0.091	0.089	0.089	0.089	0.114	0.083	0.065	0.060	0.277	0.203	0.161	0.144
Shizuoka	0.053	0.053	0.052	0.052	0.098	0.096	0.096	0.096	0.126	0.089	0.069	0.063	0.305	0.217	0.170	0.150
Aichi	0.062	0.061	0.060	0.059	0.112	0.108	0.109	0.108	0.179	0.123	0.098	0.091	0.429	0.301	0.239	0.216
Mie	0.070	0.069	0.068	0.069	0.129	0.125	0.125	0.125	0.160	0.119	0.095	0.087	0.390	0.291	0.234	0.209
Shiga	0.056	0.054	0.054	0.053	0.101	0.097	0.098	0.097	0.143	0.106	0.083	0.075	0.346	0.256	0.202	0.178
Kyoto	0.080	0.079	0.078	0.078	0.145	0.140	0.141	0.140	0.191	0.133	0.104	0.098	0.464	0.327	0.256	0.233
Osaka	0.070	0.070	0.068	0.068	0.127	0.123	0.124	0.123	0.193	0.127	0.101	0.095	0.465	0.314	0.247	0.226
Hyogo	0.062	0.061	0.060	0.060	0.114	0.110	0.110	0.109	0.154	0.107	0.083	0.076	0.374	0.262	0.203	0.180
Nara	0.068	0.067	0.067	0.067	0.125	0.121	0.121	0.121	0.162	0.109	0.082	0.075	0.390	0.268	0.202	0.180
Wakayama	0.060	0.060	0.059	0.059	0.110	0.108	0.108	0.108	0.122	0.094	0.076	0.071	0.299	0.230	0.189	0.171
Tottori	0.096	0.096	0.097	0.096	0.178	0.174	0.175	0.176	0.181	0.147	0.119	0.109	0.451	0.353	0.292	0.260
Shimane	0.095	0.096	0.096	0.096	0.177	0.174	0.175	0.176	0.163	0.134	0.112	0.106	0.403	0.322	0.277	0.254
Okayama	0.091	0.091	0.090	0.090	0.166	0.162	0.162	0.163	0.190	0.147	0.120	0.115	0.466	0.358	0.294	0.275
Hiroshima	0.077	0.077	0.076	0.076	0.140	0.137	0.138	0.138	0.173	0.130	0.104	0.099	0.421	0.315	0.255	0.236
Yamaguchi	0.081	0.082	0.082	0.081	0.150	0.147	0.148	0.148	0.158	0.120	0.097	0.093	0.387	0.292	0.239	0.221
Tokushima	0.097	0.096	0.096	0.096	0.180	0.176	0.175	0.177	0.192	0.151	0.119	0.109	0.471	0.363	0.294	0.261
Kagawa	0.083	0.083	0.083	0.083	0.154	0.151	0.151	0.152	0.170	0.134	0.107	0.102	0.416	0.322	0.264	0.242
Ehime	0.058	0.057	0.057	0.056	0.107	0.104	0.104	0.104	0.115	0.088	0.069	0.063	0.283	0.214	0.171	0.151
Kochi	0.094	0.094	0.093	0.093	0.174	0.170	0.171	0.172	0.168	0.133	0.106	0.099	0.418	0.324	0.263	0.237
Fukuoka	0.100	0.099	0.098	0.098	0.181	0.177	0.177	0.178	0.242	0.180	0.141	0.130	0.590	0.437	0.347	0.312
Saga	0.114	0.113	0.113	0.113	0.210	0.205	0.203	0.205	0.233	0.186	0.149	0.134	0.575	0.448	0.369	0.323
Nagasaki	0.090	0.090	0.090	0.090	0.167	0.164	0.164	0.164	0.181	0.139	0.109	0.096	0.445	0.337	0.270	0.231
Kumamoto	0.110	0.110	0.111	0.111	0.201	0.198	0.199	0.200	0.214	0.170	0.139	0.127	0.528	0.411	0.342	0.305
Oita	0.089	0.089	0.089	0.089	0.164	0.161	0.161	0.162	0.174	0.133	0.106	0.099	0.428	0.324	0.262	0.236
Miyazaki	0.100	0.099	0.100	0.100	0.184	0.181	0.181	0.182	0.199	0.151	0.119	0.106	0.488	0.366	0.292	0.256
Kagoshima	0.093	0.091	0.092	0.092	0.171	0.169	0.167	0.169	0.174	0.140	0.112	0.098	0.431	0.337	0.276	0.235
Okinawa	0.122	0.117	0.115	0.115	0.216	0.212	0.209	0.209	0.338	0.257	0.203	0.171	0.817	0.612	0.492	0.409
Mean	0.075	0.074**	0.074**	0.073**	0.138	0.134**	0.134**	0.134**	0.166	0.124**	0.098**	0.089**	0.406	0.301**	0.241**	0.213**

The Wilcoxon Signed-Ranks Test with the Bonferroni correction was employed for comparisons between 2015 and other time points

**p < 0.001

### Statistical analysis

Because the Shapiro–Wilk test did not confirm the normality of the data distribution, the Wilcoxon Signed-Ranks Test with the Bonferroni correction was employed for comparisons between 2015 and other time points. A value of *p* < 0.05 was considered significant. The data analysis was performed using R for Windows, Version 3.6.3 (The R Foundation for Statistical Computing, Vienna, Austria) [11].

## Results

**Fig. 1:**
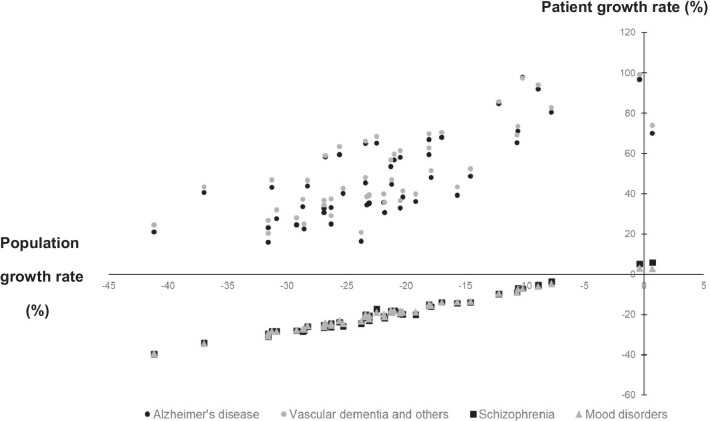
The relationship between population and patient growth rate from 2015 to 2045 in each prefecture

Table 1 displays forecasts of the number of psychiatrists and patients with mental illness in each prefecture. The mean numbers of psychiatrists and patients with schizophrenia and mood disorders in 2025, 2035, and 2045 are significantly lower than those in 2015. In each prefecture, excluding Tokyo, the number of psychiatrists is forecasted to decrease. Similarly, the number of patients with schizophrenia or mood disorders in each prefecture, excluding Tokyo and Okinawa, will decrease by 2045. On the other hand, the mean numbers of patients with vascular dementia and Alzheimer’s disease at the abovementioned three time points will be significantly higher than those in 2015. In all prefectures, the number of patients with vascular dementia or Alzheimer's disease is projected to increase by 2045. Figure 1 shows the relationship between population growth rate and patient growth rate from 2015 to 2045 in each prefecture. We also summarized the number of psychiatrists per patient (Table 2). The mean number of psychiatrists per patient for patients with schizophrenia, mood disorders, vascular dementia, and Alzheimer’s disease at the abovementioned three time points is projected to be significantly lower than in 2015.

**Fig. 2:**
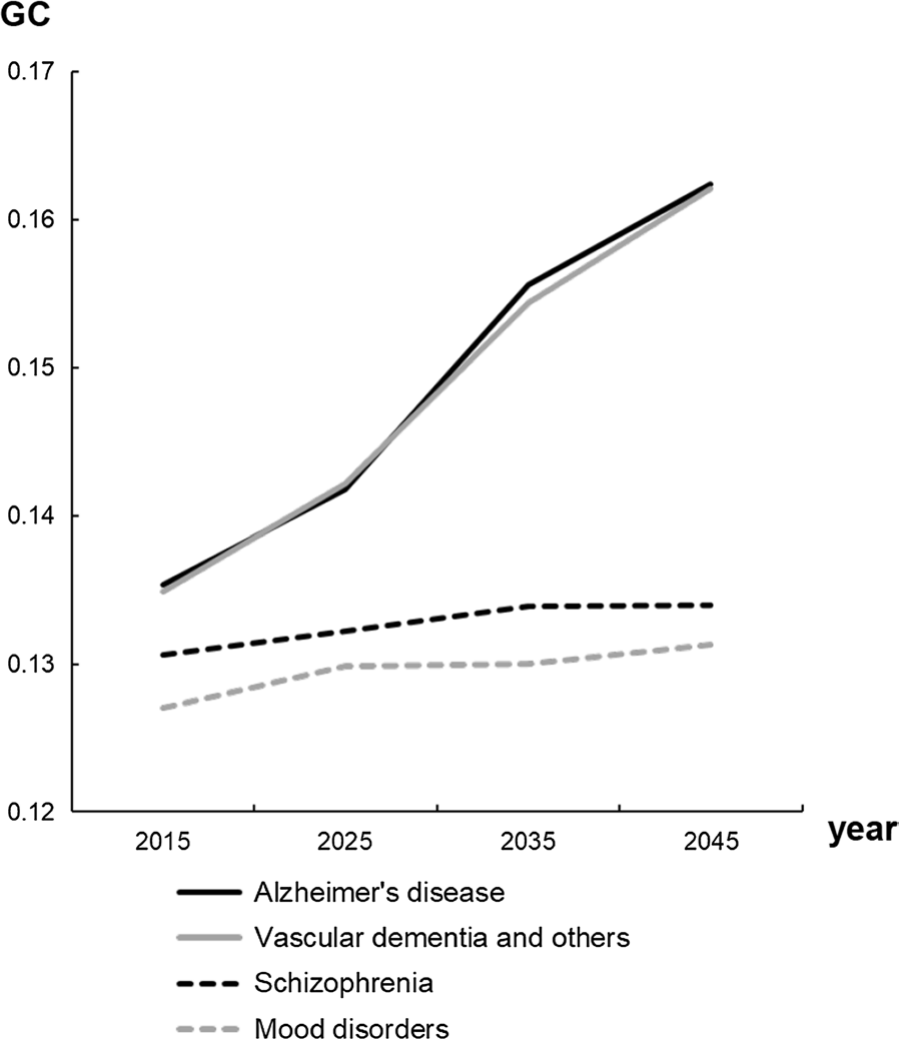
Forecasted Gini coefficients. *GC* Gini coefficient

**Fig. 3:**
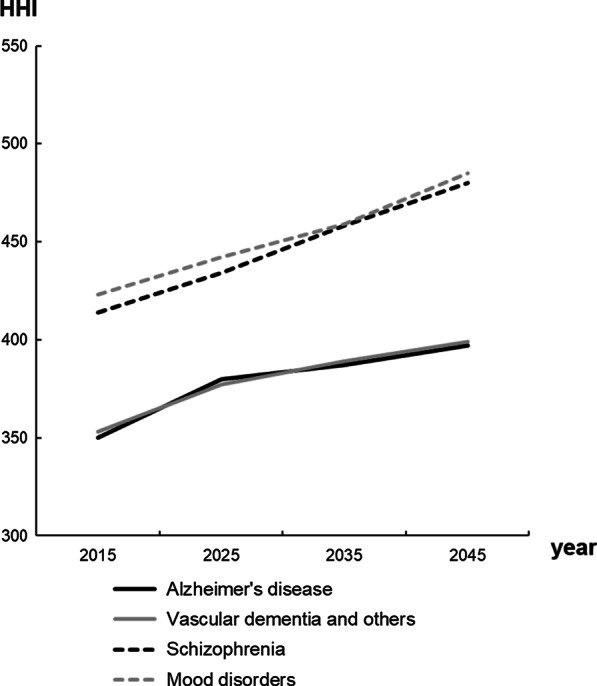
Forecasted Herfindahl–Hirschman Index. *HHI* Herfindahl–Hirschman Index

The GC and HHI for each mental illness are shown in Figs. 2 and 3, respectively. The results show that both the GC and HHI for the four mental illnesses will increase.

## Discussion

In this study, we predicted psychiatrists’ distribution for patients with mental illness in Japan. On the supply side, the mean numbers of psychiatrists in 2025, 2035, and 2045 are significantly lower than those in 2015. On the demand side, in line with depopulation, the mean numbers of patients with schizophrenia and mood disorders are significantly lower than those in 2015. However, regarding vascular dementia and Alzheimer’s disease, the mean numbers of patients with these diseases at the abovementioned three time points are significantly higher than those in 2015. For all of the abovementioned diseases, the HHI will consistently increase from 2015 to 2045. Regarding the supply–demand balance, the mean number of psychiatrists per patient for patients with schizophrenia, mood disorders, vascular dementia, and Alzheimer’s disease at the abovementioned three time points is significantly lower than in 2015. For all of the abovementioned diseases, the GC will consistently increase from 2015 to 2045.

In Japan, the shortage of physicians, including psychiatrists, has recently become a serious public health issue [12, 13]. Several studies have indicated that the cause of this shortage is related not only to the absolute number of physicians but also to their maldistribution [14, 15]. Regarding the mental healthcare system in Japan, the absolute number of psychiatrists increased from 1996 to 2012, while the GC based on the number of physicians per population did not change during the same period [16]. Because the population decline has continued to accelerate since the population peaked at 128 million in 2008 [17], we could not predict the future demand–supply balance and equality based on this short observation period. Furthermore, different patterns of healthcare services utilization for each mental illness were not considered in the analysis, and changes in the population structure might not be consistent with the utilization patterns of patients. A study from the US [18], in which the population is predicted to increase in the future, indicated that a shortage of psychiatrists per population will occur despite the increasing number of psychiatrists. Apart from mental illness, Ishikawa and colleagues forecasted the distribution of physicians for patients with acute myocardial infarction, cerebral stroke, and all medical care in Hokkaido [5]. Their results indicated that the GCs for the abovementioned three conditions will decrease from 2015 to 2035, while the HHIs will increase in Hokkaido.

Our results indicate that the change in disease structure with the increase in patients with dementia and decrease in those with schizophrenia and mood disorders will continue until 2045. Unlike the overall trend, the predicted number of patients with schizophrenia or mood disorders had not decreased in Okinawa and Tokyo by 2045. The high birth rate of Okinawa, and the migration of young people to Tokyo, might explain these predictions. The mean number of psychiatrists per patient with mental illness, especially dementia, is predicted to decrease in the same period. The maldistribution of psychiatrists will worsen in the future. To overcome this inequity, policy makers should make plans for not only the adequate geographical allocation of healthcare resources, but also the shifting of responsibilities from psychiatrists to other mental health workers. The use of information and communication technologies (ICTs) for the delivery of health services to rural communities and improved productivity of psychiatrists with more effective interventions would also ameriolate the inequity.

Several limitations of this study should be acknowledged. First, our study focuses on the number of psychiatrists as the supply side of the mental healthcare system. However, human resources in the healthcare system consist of not only psychiatrists but also nurses and other health care professionals. Furthermore, the accessibility, number and performance of medical facilities are also important factors for the supply side of the system. Analysis of supply and demand in view of these various factors is important for carrying out a more detailed analysis that will be useful for supporting policy formulation. Increasing data collection on relevant values will minimize the limitations in this area. Second, we estimated the number of psychiatrists using population projections until 2045 and psychiatrists' distribution in 2015. Our results indicate that the shortage of psychiatrists will continue to worsen if psychiatrists are allocated at the current population-to-psychiatrist ratio. However, the age distribution, retirement patterns, and future supply of psychiatrists could affect the future number of psychiatrists. Further updating research is needed to predict the number of psychiatrists for forecasting the supply–demand balance accurately. Third, our results are limited by the fact that the utilization-based approach is based on several assumptions, as with other modeling methods. The utilization-based approach could result in an over-estimation of the demand, particularly in service areas open to supply-induced demand (for instance private psychiatry services) or areas where best practices are poorly implemented. The assumption of this approach is that patients’ behavior will not change during the forecast period. Several factors, such as innovations in preventive medicine, screening and treatment, changes in medical care preferences, and changes in the capacity of the population to pay for services, could affect the behaviors of patients with mental illness. Although this analysis is based on a fixed value for the utilization rate, future research with newer rates would enable us to provide more accurate results.

In conclusion, this study forecasts the psychiatrists' distribution for patients with mental illness to analyze the healthcare supply–demand balance based on a utilization-based approach. While the number of patients with schizophrenia or mood disorders in each prefecture, excluding Tokyo and Okinawa, will decrease by 2045, the number with Alzheimer's disease or vascular dementia in all prefectures is projected to increase. For the four mental illness estimated considered, the difference between prefectures in the minimum and maximum number of psychiatrists per patient were approximately 2-folds or more in 2015. As long as psychiatrists are allocated at the current population-to-psychiatrist ratio, the shortage of psychiatrists will continue to worsen in the future. To overcome this inequity, it is necessary to discuss incentives for medical services in rural area, or mandatory requirement of practice in rural areas for psychiatrists who become board-certified psychiatrist. Although this analysis is based on a fixed value for the utilization rate, future research with frequent model updating would yield more accurate results.

## Data Availability

The datasets used in this study are freely available from the Ministry of Health, Labor, and Welfare in Japan (contact via https://www.mhlw.go.jp/toukei/sonota/chousahyo.html) and the National Institute of Population and Social Security Research (contact via http://www.ipss.go.jp/index-e.asp) for researchers.
